# Buzzing with Intelligence: Current Issues in Apiculture and the Role of Artificial Intelligence (AI) to Tackle It

**DOI:** 10.3390/insects15060418

**Published:** 2024-06-04

**Authors:** Putri Kusuma Astuti, Bettina Hegedűs, Andrzej Oleksa, Zoltán Bagi, Szilvia Kusza

**Affiliations:** 1 Centre for Agricultural Genomics and Biotechnology, Faculty of Agricultural and Food Sciences and Environmental Management, University of Debrecen, 4032 Debrecen, Hungary; astuti@agr.unideb.hu (P.K.A.); hegedus.bettina@agr.unideb.hu (B.H.); bagiz@agr.unideb.hu (Z.B.); 2 Doctoral School of Animal Science, University of Debrecen, 4032 Debrecen, Hungary; 3 Department of Animal Breeding and Reproduction, Faculty of Animal Science, Universitas Gadjah Mada, Yogyakarta 55281, Indonesia; 4 Department of Genetics, Faculty of Biological Sciences, Kazimierz Wielki University, 85-090 Bydgoszcz, Poland; olek@ukw.edu.pl

**Keywords:** *Apis mellifera*, artificial intelligence, beekeeping, climate change, machine learning

## Abstract

**Simple Summary:**

Worldwide, honeybees (*Apis mellifera* L.) are involved in pollinating both wild and economically useful plants, while their products are also used by the food and pharmaceutical industries. But currently, apiculture is encountering the adverse effects of global climate change, including more variable rainfall, shifting seasonal precipitation, and increasing temperature averages. These changes threaten the sustainable future of apiculture as these anomalies have already contributed significantly to the economic downturn of the apiculture industry in recent years. In this review, we provide an overview of the current challenges faced by apiculture due to climate change, as well as artificial intelligence (AI) applications in apiculture that can assist to address them. AI has been utilized in various scientific aspects of apiculture, such as managing hives, maintaining health, detecting pests and diseases, monitoring habitats, and managing population distribution. This is achieved by analyzing data objects such as text, audio, images, videos, sensor readings, and numerical values to investigate, model, predict, and make supporting decisions. Several shortcomings of the existing AI application are identified in this review, and the knowledge gaps regarding the development of autonomous intelligent systems for sustainable beekeeping are also highlighted.

**Abstract:**

Honeybees (*Apis mellifera* L.) are important for agriculture and ecosystems; however, they are threatened by the changing climate. In order to adapt and respond to emerging difficulties, beekeepers require the ability to continuously monitor their beehives. To carry out this, the utilization of advanced machine learning techniques proves to be an exceptional tool. This review provides a comprehensive analysis of the available research on the different applications of artificial intelligence (AI) in beekeeping that are relevant to climate change. Presented studies have shown that AI can be used in various scientific aspects of beekeeping and can work with several data types (e.g., sound, sensor readings, images) to investigate, model, predict, and help make decisions in apiaries. Research articles related to various aspects of apiculture, e.g., managing hives, maintaining their health, detecting pests and diseases, and climate and habitat management, were analyzed. It was found that several environmental, behavioral, and physical attributes needed to be monitored in real-time to be able to understand and fully predict the state of the hives. Finally, it could be concluded that even if there is not yet a full-scale monitoring method for apiculture, the already available approaches (even with their identified shortcomings) can help maintain sustainability in the changing apiculture.

## 1. Introduction

The honeybee (*Apis mellifera* L.) possesses significant economic value in both agricultural and natural ecosystems due to its role as a natural pollinator. The species’ characteristics and geographic spread are significantly influenced by climatic factors, such as variations in rainfall and temperature across the seasons [1]. Currently, we are witnessing substantial shifts in global climate patterns, and these changes are projected to persist in the future. It is anticipated that these changes will impact the diversity of plants and animals [2], including bees, as has been simulated by many researchers from various climatic conditions under numerous climatic change scenarios [3,4,5]. A study conducted by Pardee et al. [6] highlighted that climate change might induce the shift in distribution and decline in the population of the bee pollinator community, resulting in a shifting of genetic diversity, e.g., in Latin America, it was estimated to be reduced by 65% by 2050 [7]. Furthermore, this situation will lead to further implications of the primary factors contributing to the decline in bee biodiversity, such as habitat loss or fragmentation, the emergence of invasive species, the spread of diseases, and the use of pesticides [8]. Furthermore, bees exhibit certain changing behaviors that help in maintaining homeostatic circumstances in the face of climate change, both at the individual and colony levels. This poses additional challenges for the practice of apiculture management [9].

In order to develop effective management strategies, it is crucial to understand the adaptations that bees experience in response to climate change. Nevertheless, as the worldwide climate change phenomena intensify and become less predictable, comprehending the adverse consequences of climate change and developing an effective plan to alleviate them gets increasingly complicated. Artificial intelligence (AI) and machine learning (ML) could be some of the options. AI refers to the capacity of machines to acquire knowledge from past experiences, adapt to new information, and carry out tasks that resemble human abilities. It also offers innovative opportunities for identifying patterns in a vast amount of unstructured data, including the implementation of self-learning new algorithms [10]. In this review, we compile the recent AI studies in apiculture to help guide how to improve beekeeping management and gather around the cause of sustainable apiculture in the face of climate change.

We conducted a literature review, which aims to offer a comprehensive summary of the present collection of the literature related to AI application in beekeeping without any strict and predefined methodology. However, for easier literature collection, we implemented a systematic search strategy to identify the relevant literature across electronic databases, including Google Scholar, PubMed, Scopus, and Web of Science. The search was conducted without the specification of a time frame. It included variations and combinations of keywords related to artificial intelligence (e.g., “machine learning”, “deep learning”, “neural networks”) and apiculture (e.g., “*Apis mellifera*”, “beekeeping”, “honeybees”). The Boolean operator “AND” was used to combine search terms appropriately.

The inclusion criteria for studies were as follows: (1) studies focusing on applying artificial intelligence techniques in apiculture practices or beekeeping management; (2) studies examining the impact of artificial intelligence on sustainability outcomes in apiculture, including environmental, economic, and social dimensions; (3) studies published in peer-reviewed journals or conference proceedings; (4) the article is in a form of an original article, not a review. The exclusion criteria were as follows: (1) studies not directly related to artificial intelligence applications in apiculture or sustainability outcomes; (2) studies published in languages other than English; (3) studies lacking full-text availability or access to sufficient data for review.

## 2. Apiculture and Its Challenges

### 2.1. Population Reduction and Distribution

The global honeybee population is at risk due to the impacts of climate change, which include more frequent and severe extreme weather events such as heatwaves, droughts, and unpredictable rainfall [11]. Increased temperatures during warm and arid summers can lead to higher mortality rates among bee populations, particularly if the temperatures exceed the thermoregulation threshold specific to each bee species [12]. The genotype and environment significantly affect colony development and adaptation, according to Hatjina et al. [13], as *Apis mellifera* L. bee colonies found in warmer regions of southern Europe have fewer worker bees compared to colonies in colder regions. Similarly, colonies in colder areas have smaller brood populations, indicating that bees have shorter lifespans in warmer climates and a shorter period of raising broods in colder climates. Moreover, the preceding year’s warmer and drier climatic conditions are linked to a rise in winter honeybee colony mortality.

In addition to honeybee colony mortality, climate imposes a more significant influence on vegetation, hence impacting the honeybee foraging season, colony development, and overall vitality of the colony. Drought reduces soil moisture, causing water stress in plants. Consequently, flower production is diminished, resulting in limited availability of nectar and pollen resources for honeybees [12,14]. For example, a case was reported from Mediterranean areas during the drought season in 2016 and 2017 by Flores et al. [15] and reveals a significant decrease in field availability of food, which in turn increases stress levels owing to food scarcity and ultimately leads to starvation-related deaths of the *A. mellifera* L. honeybee colonies. Furthermore, they discovered a 15% reduction in the amount of Eucalyptus pollen grains in the honey, accompanied by an increase in the quantity of pollen from drought-tolerant flowers. Consequently, this resulted in a decline in the market value of the honey.

### 2.2. Genetic Diversity Reduction

Gene flow and, ultimately, shifting subspecies ranges in an area are caused by adaptation to climatic conditions. According to a study conducted in Serbia published by Tanasković et al. [16], it has long been known that warmer regions of the country are occupied mainly by *A. m. macedonica* and colder regions by *A. m. carnica* due to its adaptability. However, recent findings indicate that *A. m. macedonica* is not anymore present in Serbia. Through the examination of 14 microsatellite loci, it has been determined that Serbia now possesses a distinct hybrid honeybee population, resulting in genetic homogeneity and the formation of an admixture population. These conditions are harmful since, in honeybees, a high level of genetic diversity within a colony is necessary to boost its fitness, making it more productive, better able to maintain homeostasis, and less susceptible to disease [17]. Hungarian beekeepers have also documented this type of genetic admixture; in the past, *A. m. carnica* was considered as their indigenous subspecies. However, mitochondrial DNA and microsatellite analyses revealed a small amount of genetic introgression from other subspecies, including *A. m. mellifera* and *A. m. ligustica* [18]. In addition, the hybridization with African bees has become a growing concern for European beekeepers. It is quite alarming, as we can witness the events that occurred on the American continent. In 1956, bees (*A. m. scutellata*) imported from East Africa began to spread from southern Brazil and hybridize with already established European subspecies, leading to the emergence of a highly invasive and aggressive honeybee hybrid population [19]. Similar incidents can occur in Europe due to the consistent fluctuations in climate and changing patterns of subspecies migration. Evidence was reported in the Iberian Peninsula that shared haplotypes between bees from the African and European lines (M79 and M79a) and was detected using molecular genomics tools [20], while in another case in East–Central Europe, Oleksa et al. [21] reported that 1.64% of their bees had African mitochondrial DNA in their genetic background.

The climatic aspects can affect the distribution of the different Africanized subspecies, as they reported in Argentina; European morphotypes were found in central and southern regions, while the northern region mostly contained bees with African morphotypes [22]. This indicates that the exchange of genes between the two honeybee species is imbalanced, perhaps due to the dominance of African genetic material over European genetic material. Additionally, the African honeybees were more capable of adapting to the climate of concern [23].

### 2.3. Pest and Disease Occurrence

Parasitic, nonparasitic, omnivorous, and pollen-feeding mites are all possible inhabitants of honeybee colonies, and the prevalence of this risk increases due to unpredictable weather conditions. The honey beekeeping sector is vulnerable to infestation by several mite species, the most economically relevant of which are *Varroa destructor*, *Acarapis woodi*, *Tropilaelaps clareae* [24], *Paenibacillus larvae* [25], and *Nosema ceranae* [26]. Several cases and investigations have been documented regarding changing patterns of occurrence of various diseases as a result of global climate change. Beekeepers in Piedmont, northwest Italy, have reported about the high infestation and continuing *V. destructor* reproduction caused by mild winters, as reported in the study by Vercelli et al. [27]. The investigation conducted by Rowland et al. [28] on the climatic influences affecting prevalent honeybee pathogens in England and Wales indicates a positive correlation between the prevalence of *V. destructor* and associated diseases and increasing temperatures while suggesting a negative correlation with higher levels of rainfall and wind. As temperatures rose, the likelihood of the sacbrood virus also increased, as well as the chalkbrood disease that is caused by a fungal pathogen. Interestingly, this disease has an inverse correlation with temperature, meaning that it is more likely to occur as temperatures decrease. The modeling of the potential global distribution of the *Galleria mellonella* pest by Hosni et al. [29] indicates that the annual mean temperature and temperature annual range account for 64.2% and 19% of the distribution of pests, respectively. According to their predictions, the climate anomalies’ phenomena will cause the spread of the event to further locations and result in a more substantial financial impact on the honeybee industry in the future. Another significant threat to honeybee colony health is the small hive beetle, *Aethina tumida*, originating from southern and Central Africa but progressively spreading to all continents. The species emerged in North Africa and South Europe, and its distribution appears to be linked to climate change. The increasing temperature could potentially foster a favorable habitat for its proliferation [30]. Because of this, a study [31] predicted these climate change effects with the shared socio-economic pathways to see where they will spread with the predicted temperature changes. The countries are strongly encouraged to develop monitoring systems for *A. tumida* to prevent the further spread of the infection.

## 3. Machine Learning

Currently, we live in the Fourth Industrial Revolution (4IR) [32], wherein a vast quantity of information is accessible to us via technologies [33]. ML algorithms are effective tools for generating decision trees, rules, or statistics as they are employed to teach machines how to effectively process large databases. ML algorithms utilize a training dataset to generate rules that form a predictive model, which is validated later with a separate test dataset [34]. The outcome of the model will improve with each iteration, as it benefits from the repetition and learning process. The datasets for the algorithms can originate from many sources (e.g., IoT devices, databases) in different forms, and the outcomes of the algorithms can be evaluated using various metrics such as accuracy, precision, and speed. Furthermore, deep learning (DL) is a subset of ML that originated from the artificial neural network (ANN) but has outperformed it and is capable of performing more complex operations [35]. Several ML algorithms and models have been developed and are currently in use, each with its own specific applications in apiculture (Table 1). The following subsections concentrate on some of the primary applications of AI or ML in apiculture that address the issue of climate change. They provide a short and precise description of the experimental results, their interpretation, and the experimental conclusions that may be taken.

## 4. AI Application in Apiculture Studies

### 4.1. AI in Beekeeping Management/Hive Monitoring

**Figure 1:**
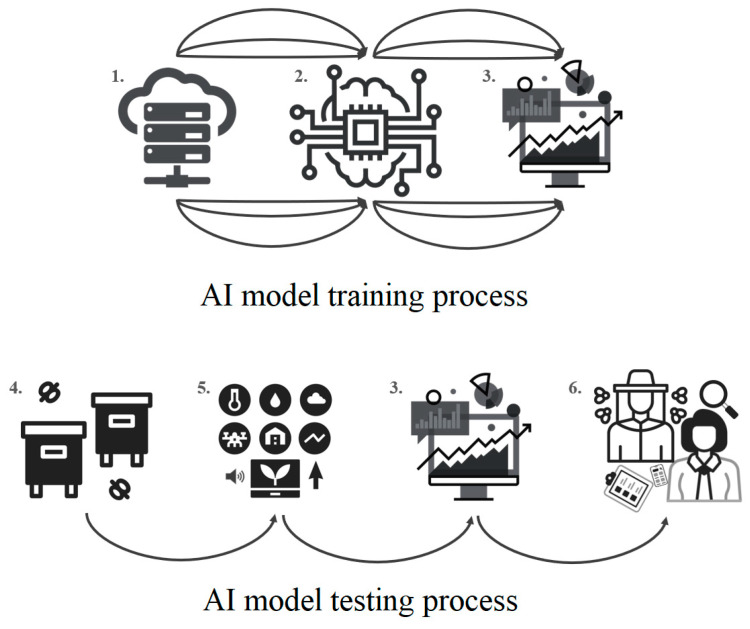
An ML-based predictive model of an AI system, with its general structure, showing the training and testing phases (1. training database; 2. ML algorithm; 3. predictive model; 4. monitoring tools; 5. test dataset; 6. practical/developmental usage of outcome).

An important issue in apiculture is the significant loss of bees due to colony collapse disorder (CCD) and the serious consequences of declining bee numbers. It could be attributed to factors such as inadequate nutrition, increased stress from ecto- and endoparasites, elevated bacterial and/or viral loads, and the combined effects of pesticides, which may ultimately be linked to climate change [86]. Comprehending the dynamics of bee colonies is intricate, and relying solely on manual inspections will not yield good outcomes for the beehives. AI technology has facilitated monitoring of beehives through several techniques such as an audio analysis [87], camera-based visual observation [88], monitoring movement [89], and analyzing physical attributes of the hives [19] (Figure 1).

Flores et al. [15] employed the Wbee system to remotely monitor the weight of beehives at fifteen-minute intervals in order to investigate the correlation between environmental conditions and hive weight. The system is structured in a three-tiered hierarchical model and relies on wireless communication. Researchers observed a correlation between decreased weight of beehives and days characterized by low temperatures, cloudiness, and rain. This suggests that the bees had limited access to food during such weather conditions. A more advanced study by Anwar et al. [90] used a hybrid deep learning model (8-sensor system—NB-IoT, LSTM) for time series forecasting and soft sensing to convert the daily variation in hive weight into predictive daily hive weight. Their result is quite sophisticated, with 83.5% of the days having mistakes of less than 25 g per frame, according to the daily estimations.

Kulyukin et al. [91] employed the BeePi system (Utah, United States of America), which monitors beehives using audio by comparing several DL and traditional ML techniques to identify audio samples from microphones positioned about 10 cm above the landing pads of Langstroth beehives. A 30 s audio file was recorded every 15 min for classification using four different ML algorithms: LR; KNN, with a linear kernel one vs. rest (SVM OVR); and RF. LR demonstrated the highest performance among others, indicating that a less complex raw audio CNN yielded the most accurate classification of the audio samples and also showed a high potential for practical use. In another study, Di et al. [87] suggested utilizing the VGGish embedding, a model for audio classification similar to the visual geometry group, in conjunction with the KNN model for audio classification in beehive audio monitoring. Additionally, Zgank [92] introduced a system that uses IoT technology based on acoustic signals to classify bee swarm activity. The system utilized feature extraction techniques such as Mel-frequency cepstral coefficients (MFCCs) and linear predictive coding (LPC) to analyze the input audio signal and monitor this significant occurrence in beehives.

In image classification, using various models of deep learning classification, Berkaya et al. [93] identified different conditions using honeybee photos captured at beehives with Deep Neural Networks (DNNs) and SVM algorithms. A variety of conditions can be identified by the suggested models, including healthy bees, pollen-bearing bees, and abnormalities including ant difficulties, small hive beetles, hive robberies, and Varroa parasites, all with a remarkably high accuracy of 99.07% and a relatively quick classification time. A recent study conducted by Williams et al. [88] examined the enhancement in thermal cameras using machine learning techniques (KNN, neural networks/NNs, RF, and SVM). Despite having inferior baseline specs compared to a competitive optical camera, these cameras were able to attain the same degree of efficiency in monitoring the activity at the entrance of a beehive. The thermal camera provides the benefit of functioning efficiently in all circumstances without requiring adequate lighting conditions. In the case of thermal data, the KNN and NN algorithms were the most efficient, while the feature specificity was the best in the case of SVM and RF. Their analysis confirms that the thermal camera effectively captured and accurately identified the flight. In their subsequent investigation, Williams et al. [83] conducted experiments to evaluate the efficacy of this thermal camera in implementing a real-time radar signal classification system with SVR algorithms for monitoring and quantifying bee activity at the hive’s entrance.

Furthermore, Alves et al. [94] developed DeepBee© software using imaging data, which can identify cells in comb photos and classify their contents into seven categories: eggs, larvae, capped brood, pollen, nectar, honey, and others. The objective is to assist beekeepers in evaluating the quantities of comb cells containing brood and food reserves, allowing the evaluation of the colony’s nutritional and health condition, queen quality, and honey production potential. By employing the Circle Hough Transform and semantic segmentation technique, a cell detection rate of 98.7% was achieved. Among 13 different CNN algorithms evaluated for comb cell classification, MobileNet emerged as the optimal option, achieving a balance between training cost and accuracy, with processing time averaging approximately 9 s per comb image and an F1-Score of 94.3%.

### 4.2. AI in Bee Health and Disease Monitoring

For effective disease management and mitigation, it is important to comprehend the patterns of dispersion and how climatic conditions impact them. By considering multiple recorded parameters, modeling systems can assist in predicting it. Using the Maximum Entropy model’s algorithm, Hosni et al. [29] forecasted the dispersal of *Galleria mellonella* pests by building the model using 19 components of bioclimatic data and the reported disease occurrence. The most effective climatological parameters that affect the dispersion of this pest were determined to be the annual temperature range and mean, along with yearly precipitation. Slovenia, Slovakia, France, Italy, Belgium, the United Kingdom, the Mediterranean coast, and a few other countries on other continents were on the list of the high-potential future habitats for this pest. The same research approach and results were employed to investigate the distribution of *A. tumida*, *Galleria mellonella*, and *Oplostomus haroldi*, besides *Varroa destructor* in Kenya [95] and Tanzania [96]. They forecasted a growing likelihood of suitable habitats for these pests in various regions of these countries in the future.

Monitoring the infestation of pests and determining their level is an important and challenging duty for beekeepers, as early detection is a high determinant in disease management. Research utilizing videos of honeybee behaviors, which are subsequently converted into image patches [97,98,99], was able to detect the parasite infection in beehives. The input photos underwent classification using diverse methodologies, such as the Bayesian theorem, statistical learning theory, and a combination of decision trees, to detect the presence of mites. The outcome revealed a high categorization accuracy exceeding 70% and resulted in time savings of over 50% compared to manually observing mite presence. In another example, Wachowicz et al. [100] propose a method for the real-time monitoring and detection of pests by analyzing a combination of various detection methods (camera-based IoT devices, pre-trained CNN approach) and a cloud data center for monitoring and notification. The study demonstrates a significant enhancement in accuracy, reaching up to 90% of parameters from the video stream of hive conditions using IoT devices, and edge-based *V. destructor*, by employing a convolutional neural network methodology. However, it is important to clarify that the detection capability of the device is limited to the mites specifically within honeybee cells, and does not extend to the mites present on the bodies of bees. Besides this, Mrozek et al. [101] used another monitoring IoT device with optical recordings (20 pictures from a video stream). The collected data were analyzed with a CNN algorithm, while the resulting information (bee and *V. destructor* identification) was stored/transferred using a cloud that the beekeeper can reach. The precision (70%) and sensitivity (90%) of the Varroa detection rate were high, as well as the honeybee identification (100% and 70%), which means that this method is also useable to prevent a strong infection of the colonies. Some other multi-sensor bee health monitoring systems have also been developed, such as IndusBee4.0 [102] and the BeePi system [103].

An ML model can also accurately assess and forecast temperature declines within honeybee colonies, a crucial determinant of colony well-being. In research by Braga et al. [64], they measured six aspects of the hive, internal temperature, internal humidity, mean fanning, mean noise, mass, and external temperature, with the Arnia system that has three sensors inside the hive: temperature, humidity, and sound sensors; besides these, there is a digital scale under the hive. The gathered data were analyzed using an ML model that employed an LSTM technique. This proposed remote hive monitoring system could predict the temperature one day in advance, with a 0.5% root mean square error (RMSE).

A study by Robles-Guerrero et al. [43] compared five different ML models, to be able to automatically assess the health status of the hive, by analyzing their acoustic data. The following were the mostly used models: the KNN, the LR, the NN, the RF, and the SVM. These are simpler than the DL models, resulting in faster and easier training, but still require computer resources. They gathered acoustic data (by Rpi microphones) from five Carniola honeybee colonies, of which two were strong colonies, two were weaker, and one colony was queenless with the lowest bee population. It was determined that NN and SVM were the optimal alternatives due to their efficient classification time and excellent performance metrics.

### 4.3. AI in Bee’s Habitat and Climate Management

In the field of apiculture, bees and their interactions with the natural environment are integrated into social–ecological systems. Gaining a comprehensive understanding of the many interrelationships within this system has become more important for effective environmental management and the implementation of innovative interdisciplinary methods that promote sustainable beekeeping [104]. Braga et al. [98] proposed a clustering and classification algorithm (Naive Bayes, KNN, and RF) to detect seasonal patterns in honeybee behavior. The weekly and monthly meteorological data, along with the beehive weight data, were combined to detect the occurrence of swarming, determine the optimal moment for seasonal management, and assess the prevalence of pathologies. In the classification stage, they discovered six distinct seasonal honeybee patterns, each with hit rates reaching as high as 99.67%. Later, Patel et al. [105], with more complex data, utilized a spatially explicit modeling technique, a machine learning algorithm, and an agent-based model to simulate the migration of beehives in relation to the geographic distribution of bee food supply in western Australian apiculture. They effectively illustrated the variations in spatial distributions of the primary bee food species, both on a seasonal and monthly basis, as well as the overall species diversity. Based on that, they predicted the future migration pattern and recommended a change in the hives towards the east part.

It is well known that bees are not only found in their natural habitats, but they are also utilized for pollination, such as in crop fields. This is why beekeepers relocate their honeybees to these areas. However, there is an ongoing disagreement between farmers and beekeepers around the world. One of the reasons is due to frequent bee poisoning (e.g., neonicotinoids), which is usually caused by using sprays before the end of the foraging activity of the bees, leading to significant losses to the beekeeping community every year [106]. Therefore, it is important to accurately forecast the remaining duration of time that bees allocate to foraging in the fields. To overcome this issue, Torresani et al. [107] developed a non-invasive IoT-based machine learning model that considered real-time bee activity, weather, and sunset time to forecast this foraging time. The researchers demonstrated that the GBR was the best suitable regression model for effectively differentiating between foraging and other behaviors in bees. This model can be utilized by farmers to determine optimal spraying timings that are safe for honeybees. Another study [76] investigated the periods of time when bees were collecting food, measuring the number of bees foraging every half hour between 5:00 am and 4:00 pm. The aim of this was not only to determine the number of foraging insects but also the relationship between the number of these bees and the time of the day. A polynomial regression algorithm was used for data processing in this case as part of the machine learning process. The generated model was suitable for testing the inter-day variability of the data obtained, and the gained conclusions can be integrated into future research on bees as well as being possibly applicable in the field.

In a study, real colored Red–Green–Blue (RGB) images were taken by an Unmanned Aerial Vehicle (UAV) to measure flower cover and diversity by association, bee abundance, and diversity. There, Torresani et al. [108] discovered a standardized, large-scale, and cost-effective way to monitor bee habitat remotely (e.g., ‘flower cover data’). They suggested that optical pictures with a better spatial resolution yield more accurate results when using RF machine learning algorithms instead of NN and SVM. The environment of bees can influence the composition of the royal jelly they produce, as demonstrated in scientific research released in 2023 [105]. Because of this, ML methods can even be used to monitor the quality of royal jelly production, as demonstrated in this study. Here, they showed that combining stable isotopes with artificial ANN models (excellent accuracy, sensitivity, and specificity) and considering the unique proven correlation between stable isotopes and environmental factors (temperature, precipitation, sunlight) can provide promising ideas for monitoring the authenticity of royal jelly.

### 4.4. AI in Subspecies Distribution and Population Management

There are different kinds of *A. mellifera* L. subspecies, such as previously mentioned *A. m. ligustica* (Italian honeybee), *A. m. carnica* (Carniolan honeybee), *A. m. mellifera* (dark European honeybee), and *A. m. scutellata* (African honeybee). New methods based on genetics can help to study their geographic distribution more accurately, but it is still mainly performed based on morphometry [22]. Conventional approaches of taxonomic examination are laborious and time-consuming, encouraging people to seek a more efficient solution. In a publication by De Nart et al. [48], they researched the use of AI with ML techniques based on CNN to recognize honeybee subspecies. The research was conducted using four CNN models, utilizing a collection of 9887 wing pictures from seven subspecies and the commonly used Buckfast hybrid. From them, the Inception ResNet V2 performed the best (higher than 98% for accuracy, precision, and specificity), which also surpassed the performance of the traditional method. This demonstrated that an automatic image recognition (with only wing pictures) and ML technology is definitely a promising solution for bee species recognition, promoting the preservation of the biodiversity of honeybee species and to preserve pure lines for the market.

With a similar aim, Rodrigues et al. [84] developed DeepWings© software, which analyzed 19 landmarks in a right forewing image, to make a fully automated morphometric-based assessment of honeybee subspecies. They started to work with a smaller but richer dataset including 7634 forewing images, containing pictures of 26 subspecies. In this study, they also used CNN as a wing detector, but for detecting the 19 features, they applied U-Net DLtool, and SVM to classify subspecies. Eventually, they successfully processed 10 images within a time frame of 14 s, which is the minimum acceptable duration. Initially, the accuracy rate was 86.6%. However, after training with just five subspecies, the precision improved to 95.8%. It is possible that this percentage might have further risen with further training over time. This software has 19 comprehensive features, which can also make a significant addition to other similar investigations. This same software was used in another study [109], with 14,816 wing images from 2601 colonies that belonged to three Apis mellifera subspecies. The results were somewhat similar to the first accuracy of the previous paper, with 89.7% for the *A.m. iberiensis* (Iberian honeybee), and 88.3% for the Carniolan honeybee, but in the case of the dark honeybee, they only received a 41.1% match. It has been demonstrated that while the DeepWings© software is a valuable tool for identifying subspecies, it can still cause confusion in identifying subspecies, particularly when the honeybees have genetic impurities.

In addition to a remote optical analysis, acoustic signals can also be utilized for population management by automating the identification of colonies that are ready to swarm. Dimitrios et al. [110] made a comparison between three different classification algorithms: the KNN and SVM, and their new U-Net CNN. The acoustic data that were studied from five colonies, for 5 months, twice a day, for 6–6 h, were acquired by IoT devices specifically designed for this research, which include microphones, temperature sensors, and humidity sensors. Among the various ML models used, KNN and SVM demonstrated the highest accuracy in identifying early and late swarming. However, when it came to early detection specifically, SVM outperformed the rest, with the U-Net CNN method performing similarly.

Certain research endeavors to build a hybrid insect society, in which honeybees and autonomous robots engage in mutual interaction. In order to carry out this, the robots must be fine-tuned using evolutionary algorithms, which necessitate the evaluation of population density and behavioral data. To be able to carry out this, Salem et al. [111] used Combined Actuator Sensor Units (CASUs), which include different sensors, and could communicate with the bees through their heat, movement, etc. They used three new algorithms (J48 Decision Tree—C4.5 based on ID3; JRip Rules Classifier—RIPPER; PART—developed version of C4.5 and RIPPER algorithms) with different setups to learn from the detected information. At the end of the study, the PART model performed the best in both accuracy and ruleset size, while the worst was the JRip. With this, they were able to estimate the bee density accurately. This method can be an option to be used in other future experiments with bee–robot interactions, allowing for control over the colonies.

## 5. Conclusions and Direction towards Sustainable Agriculture

This review provides an in-depth survey of techniques based on AI and ML that are being used to overcome the difficulties that beekeepers face in coping with climate change. In the current era of the Fourth Industrial Revolution, machine learning algorithms have become increasingly accessible. These algorithms undergo continual training using the data they receive, and they have a diverse variety of applications. Due to this capability, they are able to process a wide range of data, as demonstrated in the setting of apiaries. These data can encompass various factors such as temperature, sound, pictures, video, weather conditions outside the hive, humidity, air circulation, weight, bee activity, and numerous other variables. Hence, the data can be utilized for the purpose of training and refining algorithms, or for evaluating models that have already been validated and approved. The data in the examined research were often gathered using a non-invasive technology based on the Internet of Things (IoT) or an instrument equipped with a specialized sensor or were derived from an existing dataset.

When comparing methodologies, SVM and NN algorithms are frequently utilized and generally considered the most suitable, with CNN being one among them. The NN models usually had an accuracy of over 95%, and they were used mostly in studies too. Besides these, they are the basis for DL technologies. When the ideal database had been discovered and the logarithm was effectively chosen and tested, numerous investigations employed a completely automated monitoring system utilizing ML, such as BeePi (monitoring beehives through audio), the DeepBee© system (monitoring the quality of combs by pictures), and the DeepWings© software (monitoring the morphology by pictures). Therefore, the ML tools are currently functional, albeit not as a comprehensive monitoring technology of the apiculture, due to their ongoing development. These methods can help in maintaining sustainability in the hives despite our changing environment, by monitoring the colonies for a possible early intervention of the beekeeper.

Despite the numerous approaches developed by researchers, the continuous advancement in technology necessitates the processing of larger volumes of data within increasingly shorter timeframes. According to the studied publications, it has been seen that in some cases, the amount of data being processed is still low, and there are still inaccuracies in certain algorithms, especially with genetic purity. Thus, this can provide a challenge as increasingly intricate approaches necessitate the utilization of many algorithms for ML-based solutions (e.g., CNN and SVN in DeepWings software). In addition, the training dataset needs to be enriched and augmented to allow the algorithm to start with a sufficient reference, thus achieving faster process time, higher precision, and minimal computational consumption in the first use. Consequently, it is necessary to enhance and optimize the monitoring devices in order to obtain a wide range of data, enabling a comprehensive evaluation of the colonies. But in the end, every ML monitor system development needs to be tested faster in farm-setting conditions preferably in an apiary to ensure its accessibility and applicability to the beekeepers.

## Figures and Tables

**Table 1:** Summary of the machine learning algorithms used in the beekeeping studies used in this review.

Model	Short Description	Usage in Honeybee Research
Artificial Neural Network (ANN) and Neural Networks (NNs)	As computational models inspired by the structure and function of the human brain, ANNs and NNs comprise interconnected nodes, or neurons, arranged in layers. ANNs are renowned for their capacity to discern intricate patterns and relationships within data, rendering them applicable across diverse domains [36]. NNs require less formal statistical training, can detect complex nonlinear relationships between dependent and independent variables, have all possible interactions between predictor variables, and have the availability of multiple training algorithms [37].	Monitoring of pesticide effect on bee behavior [38]. Modelling the flight activity of workers at the hive entrance [39]. Classification of honey [40]. Unraveling associations between the environment and oxidative stress biomarkers in honeybees [41]. Determining daily performance of colony based on weather [42]. Classifying bee colony acoustic patterns [43]. Characterizing seasonal patterns of colony development [44].
Convolutional Neural Network (CNN)	It is widely employed in image and video recognition tasks, which automatically learn relevant features from raw input data, making them highly effective in tasks such as image classification, object detection, and image segmentation [45].	Estimation of honeybee density in hives [46]. Decoding waggle dances [47]. Honeybee subspecies determination using image recognition for honeybee wing analysis [48].
Extremely Randomized Trees (ETs)	A type of ensemble learning method that constructs several decision trees to perform classification or regression tasks, with the aim to provide additional randomness into the process of constructing trees in order to enhance generalization and mitigate overfitting [49].	Bee sound classification for hives management [50]. Queen bee detection from audio recording [51].
Validated Counter-Propagation Artificial Neural Network (CPANN)	A specialized variant of ANNs that integrate elements of counter-propagation networks with validation techniques, and typically comprises two layers: an input layer and a competitive layer. This process enables CPANN to cluster data into meaningful groups or classes based on similarities in input patterns; it also incorporates validation procedures to optimize network performance and enhance generalization capabilities [52].	Classification models for substances exhibiting acute toxicity for honeybees [53].
Gradient Boosting Regressor (GBR)	Mainly used for regression problems, by making predictions using outputs from multiple decision trees. GBR constructs one tree at a time and corrects the errors of the preceding trees [54].	Identifying factors influencing queen body mass [55]. Prediction of honey harvest [56]. Revealing the relationship between number of bees in the beehive and temperature [57].
K-Nearest Neighbor (KNN)	A straightforward ML algorithm utilized for classification and clustering tasks by assessing the proximity of data points to categorize or predict the grouping of individual observations. For each new observation, KNN determines classification by computing its distance from all known observations. The majority class of the K-nearest neighbors then determines the classification outcome [58].	Discrimination of unifloral honeys [59]. Classifying bee colony acoustic patterns [43].
Logistic Regression (LR)	Used for modeling binary or categorical outcomes by predicting the probability of a categorical outcome based on one or more predictor variables. It can be used for both classification and regression problems, but it is more commonly used for classification [60].	Classifying honeybee sounds with spectrogram features [61]. Classifying bee colony acoustic patterns [43].
Long Short-Term Memory (LSTM)	A type of ANN designed to process sequential data by maintaining an internal state or memory. It can handle long time-series data, can avoid vanishing gradient problems, can handle variable-length sequences, has a memory cell that can store and retrieve information, and has gradient flow control [62].	Detection of queenlessness in beehives [63]. Forecasting sudden drops of temperature in pre-overwintering honeybee colonies [64].
Naive Bayes (NB)	NB classifier is based on the Bayes Theorem to generate the predictions for each observation by classifying a sample into a group that is most likely to have its attributes [65].	Selecting features for honeybee subspecies determination [66].
High-Dimensional Discriminant Analysis (HDDA)	Used for discriminant analysis when there are a large number of variables (features) compared to the number of observations (samples) [67].	Classification of unifloral honey [59].
Partial Least Square (PLS)	Enables the comparison of numerous response variables and multiple explanatory variables in a multivariate setting. PLS is a covariance-based statistical method that is commonly known as structural equation modeling or SEM [68].	Mineral content detection in honey [69] and bee pollen [70]. Identify honey based on its various entomological origins [71]. Honey quality prediction [72].
Penalized Discriminant Analysis (PDA) and Shrinkage Discriminant Analysis (SDA)	PDA and SDA are employed in the field of classification and pattern recognition. It is a continuation of Linear Discriminant Analysis (LDA). The primary objective of PDA is to enhance the efficacy of LDA, particularly in scenarios where there is an imbalance between the number of variables (features) and observations (samples), or when the data are affected by multicollinearity [73]. SDA aims to enhance the estimate of the covariance matrix utilized in LDA by implementing a shrinkage strategy on the sample covariance matrix [74].	Classification of unifloral honey [59].
Polynomial Regression Algorithm (PR)	A form of linear regression in which the relationship between the input variable x and the output variable y is modeled as a polynomial and considered to be a special case of linear regression [75].	Bee foraging behaviors [76].
Random Forest (RF)	Based on a group (or forest) of decision trees used to generate the classifications. Decision trees are structures that are based on decision rules to branch out into possibilities and create a path. At the end of the path is the rating assigned to the entry [77].	Predicting overwintering survival [78]. Predicting honey harvest [56]. Chemical toxicity to honeybee assessment [79]. Classifying bee colony acoustic patterns [43].
Support Vector Machine (SVM)	It can be used for classification, regression, or other tasks. It is good for producing high-quality results with interpretability and flexibility; it does not require too much memory, and is effective in high-dimensional spaces [80].	Classifying bee colony acoustic patterns [43,81]. Detecting bee queen presence [82]. Developing real-time bee counting radar [83]. Discrimination of honeybee subspecies based on wing images [84].
Support Vector Regressor (SVR)	The SVR is the regression algorithm of SVM. It can find the best fit line, which is the hyperplane that has the maximum number of points [85].	Real-time radar for bee count activity [83].

## Data Availability

No new data were created or analyzed in this study. Data sharing is not applicable to this article.
